# The Complex Action Recognition via the Correlated Topic Model

**DOI:** 10.1155/2014/810185

**Published:** 2014-01-16

**Authors:** Hong-bin Tu, Li-min Xia, Zheng-wu Wang

**Affiliations:** ^1^School of Information Science and Engineering, Central South University, ChangSha, Hunan 410075, China; ^2^School of Traffic and Transportation Engineering, ChangSha University of Science & Technology, ChangSha, Hunan 410004, China

## Abstract

Human complex action recognition is an important research area of the action recognition. Among various obstacles to human complex action recognition, one of the most challenging is to deal with self-occlusion, where one body part occludes another one. This paper presents a new method of human complex action recognition, which is based on optical flow and correlated topic model (CTM). Firstly, the Markov random field was used to represent the occlusion relationship between human body parts in terms of an occlusion state variable. Secondly, the structure from motion (SFM) is used for reconstructing the missing data of point trajectories. Then, we can extract the key frame based on motion feature from optical flow and the ratios of the width and height are extracted by the human silhouette. Finally, we use the topic model of correlated topic model (CTM) to classify action. Experiments were performed on the KTH, Weizmann, and UIUC action dataset to test and evaluate the proposed method. The compared experiment results showed that the proposed method was more effective than compared methods.

## 1. Introduction

Automatic recognition of human actions from video is a challenging problem that has attracted the attention of researchers in recent decades. It has applications in many areas such as entertainment, virtual reality, motion capture, sport training [1], medical biomechanical analysis, ergonomic analysis, human-computer interaction, surveillance and security, environmental control and monitoring, and patient monitoring systems. Human complex action recognition is an important research field of the action recognition. Among various obstacles to human complex action recognition, one of the most challenging is to deal with “self-occlusion”, where one body part occludes another. Adaptive self-occlusion behavior recognition has been traditionally tackled by applying statistical prediction and inference methods [2]. Unfortunately, basic numerical methods have proved to be insufficient when dealing with complex occlusion scenarios that present interactions between objects (e.g., occlusions, unions, or separations), modifications of the objects (e.g., deformations), and changes in the scene (e.g., illumination). These events are hard to manage and frequently result in tracking errors, such as track discontinuity and inconsistent track labeling.

Our models are motivated by the recent success of “bag-of-words” representations for object recognition problems in computer vision. The common paradigm of these approaches consists of extracting local features from a collection of images, constructing a codebook of visual words by vector quantization, and building a probabilistic model to represent the collection of visual words. While these models of an object as a collection of local patches are certainly not “correct” ones, (e.g., they only model a few parts of objects and often ignore many structures), they have been demonstrated to be quite effective in object recognition tasks [3–5]. For the recognition approaches, some structures have been lost by moving to this representation. However, this representation is much simpler than the one that explicitly models temporal structures. there has been previous work (e.g., Yamato et al. [6], Bobick and Wilson [7], and Xiang and Gong [8]) that tries to model the full dynamics of videos using sophisticated probabilistic models (e.g., hidden Markov models and dynamic Bayesian networks). Li and Perona [9] use a variant of LDA for natural scene categorization. Sivic et al. [10] perform unsupervised learning of object categories using variants of the pLSA model. In this models, the “words” correspond to local patches extracted by interest point operators, and the “topics” correspond to the different object categories. Fergus et al. [11] extend pLSA to incorporate spatial information in a translation and scale-invariant manner and apply them to learn object categories from Google's image search. Wang et al. [12] designs a simultaneous classification and annotation framework which extends from LDA and allows image feature and text word share the same dimensional topic space. Putthividhya et al. [13] propose a more general and flexible annotation model which allows different topic spaces for image feature and text word. Bissacco et al. [14] use LDA for human detection and pose classification. The “visual words” in their model are vector quantizations of histogram of oriented gradients in the training images. Niebles et al. [15] recently demonstrate some impressive results on unsupervised learning of human action categories using pLSA and LDA models for human action recognition. Wong et al. [16] adopt pLSA models to capture both semantic (content of parts) and structural (connection between parts) information for recognizing actions and inferring the locations of certain actions.

Optical flow-based action detection methods are well known [17–19]. Efros et al. [20] recognize human actions at a distance in low resolution by introducing a motion descriptor based on optical flow measurements. Ahmad and Lee [21] propose a view independent recognition method by using the C-artesian component of optical flow velocity and human body shape feature vector information. Usually optical flow is used with other features, because it is noisy and inconsistent between frames [22]. Optical flow histograms have also been used to analyze the motion of individual behavior videos. The time series of histogram of optical flow has been modeled as a nonlinear dynamical system using Binet-Cauchykernels in [23]. However, this approach cannot deal with large motion, for example, a rapid move across frames.

In order to overcome the shortcomings mentioned above, we propose an adaptive self-occlusion action recognition method that not only estimates the occlusion states of body parts but also recognizes the occlusion behavior. Firstly, the Markov random field was used to represent the occlusion state of human body parts. Secondly, the structure from motion (SFM) is used for reconstructing the missing data of point trajectories. Then, we can extract the key frame based on motion feature from optical flow and the ratios of the width and height are extracted by the human silhouette. Finally, we use the topic model of correlated topic model (CTM) for action recognition. Experiments were performed on the KTH, Weizmann, and UIUC action dataset to test and evaluate the proposed method. The experiment results have shown that the proposed method is effective in action recognition.

The reminder of this paper is organized as follows.  Section 2 presents the adaptive occlusion state estimation by Markov random field (MRF). In Section 3, we reconstruct the missing data of point trajectories by SFM (structure from motion).  Section 4 explains feature representation.  Section 5 explains algorithm of action models and the design of the classifier.  Section 6 explains the results and analysis of the proposed approach. Finally, we conclude the paper in Section 7.

## 2. The Adaptive Occlusion State Estimation

The human body is divided into 15 key points, namely, 15 joint points representing the human body structure (torso, pelvis, left upper leg, left lower leg, left foot, rightupper leg, right lower leg, right foot, left upper arm, left lower arm, left hand, rightupper arm, right lower arm, right hand, and head) [24], which represent the human body behavior. In order to calculate the observation, spatial relations, and the motion relationship, we use Markov random field (MRF), which can determine the occlusion positions of the body joints.

In this paper, we use a state variable in the Markov random field (MRF) for representing the self-occlusion relationship between body parts. The MRF is a graph *G* = (*V*, *E*), where *V* is the set of nodes and *E* was the set of edges. The graph nodes *V* represent the state of a human body part and graph edges *E* model the relationships between the parts [25]. The probability distribution over this graph was specified by the set of potentials defined over the set of edges. The MRF structural parameters [24, 25] are defined as follows:

*X*_*i*_ = (*x*_*i*_, *y*_*i*_, *z*_*i*_): the *i*th joint point coordinates; *X* = {*X*_1_, *X*_2_,…, *X*_15_}: extract the key points of the body 15, *γ*(*X*_*i*_) (*i* ≤ 15): the *i*th joints visible parts, and this parameter is used to determine occlusion relation between nodes. When occlusion occurred, trajectories intersected between

(1) $$\begin{array}{c} X_{i}\left(X_{i}\left(x_{i},y_{i},z_{i}\right)\right),\;\;X_{j}\left(X_{j}\left(x_{j},y_{j},z_{j}\right)\right); \end{array}$$

Λ = {Λ_*ij*_}  (*i* ≤ 15, *j* ≤ 15): the occlusion relation among the 15 body joints. When Λ_*i*,*j*_ = 0, the *i*th and *j*th joints are not occluded. When Λ_*i*,*j*_ = 1, the *i*th occluded *j*th. When Λ_*i*,*j*_ = −1, the *j*th occluded *i*th; *λ*_*i*_ = {*λ*_1_,…, *λ*_15_}: the *i*th occluded joints node.

We apply the MRF model presented in [24, 25] to optimally estimate potential of kinematic relationship and similar to [26] for calculating three potential functions in video activity analysis. The potential of kinematic relationship and three potential functions were defined as follows.

(1) Kinematic relationship is calculated
  (2) $$\begin{array}{c} \psi_{ij}^{K}\left(X_{i},X_{j}\right)=N\left(d\left(x_{i},x_{j}\right);\mu_{k},\delta_{K}\right)f\left(\theta_{i},\theta_{j}\right). \end{array}$$
 This function indicates the position of two adjacent joints and the angles among joints. *d*(*x*_*i*_, *x*_*j*_) is the Euclidean distance between two adjacent joints. *N*(·) is the normal distribution with *μ*_*k*_ = 0 and standard deviation *δ*_*K*_.
(2) The Potential Functions
 The observation potential function is
  (3) $$\begin{array}{c} \phi_{i}\left(I,X_{i};\wedge_{i}\right)=\phi_{i}^{C}\left(I,X_{i};\wedge_{i}\right)+\phi_{i}^{E}\left(I,X_{i};\wedge_{i}\right), \end{array}$$
 where *ϕ*_*i*_(*I*, *X*_*i*_; ∧_*i*_) is the potential of observation, *ϕ*_*i*_^*C*^(*I*, *X*_*i*_; ∧_*ij*_) is the potential of the color, and *ϕ*_*i*_^*E*^(*I*, *X*_*i*_; ∧_*i*_) is the potential of the edge, *I*: input image.

The potential of the color is

(4) $$\begin{array}{c} \phi_{i}^{C}\left(I,X_{i};\wedge_{ij}\right)=\phi_{i}^{C_{\text{visible}}}\left(I,X_{i};\wedge_{ij}\right)+\phi_{i}^{C_{\text{occluded}}}\left(I,X_{i};\wedge_{ij}\right), \end{array}$$

where the first term is *X*_*i*_ of probability of occurrence of color in the visible area and the second term is for the occluded area as follows:

 
*ϕ*_*i*_^*C*_visible_^(*I*, *X*_*i*_; ∧_*ij*_): the motion state of *X*_*i*_ (the *i*th body joint) in the viewing area;
 
*ϕ*_*i*_^*C*_occluded_^(*I*, *X*_*i*_; ∧_*ij*_): the motion state of *X*_*i*_ (the *i*th body joint) in the occluded area.

The visible term is formulated as

(5) ϕiCvisible(I,Xi;λi) =∏u∈(γ(Xi)−(γ(Xi)∩γ(Xj)))PC(Iu) =∏u∈(γ(Xi)−(γ(Xi)∩γ(Xj)))P(Iu ∣ foreground)  P(Iu ∣ background)

*P*(*I*_*u*_ | foreground) and *P*(*I*_*u*_ | background) are the distributions of the color of pixel *u* given the foreground and background:

(6) ϕiCoccluded(I,Xi;λi) =∏u∈(γ(Xi)∩γ(Xj))[zi(Iu)+(1−zi(Iu))PC(Iu)]

and *z*_*i*_(*I*) is calculated as follows:

(7) $$\begin{array}{c} z_{i}\left(I\right)=\frac{1}{N}\sum \left(\phi_{j}^{C}\left(I_{u},x_{j}\left(t\right);\lambda_{j}\right)\right); \end{array}$$

*u* ∈ (*γ*(*X*_*i*_)∩*γ*(*X*_*j*_)): the occlusion area is determined by the calculated overlapping region of *X*_*i*_ and *X*_*j*_ and *N* is the sum of all occlusion nodes. *f*(*θ*_*i*_, *θ*_*j*_) = 1, *T*_lower_ ≤ *θ*_*i*_ − *θ*_*j*_ ≤ *T*_upper_, where *T*_lower_ and *T*_upper_ are the lower and upper bound of motion area between *X*_*i*_ and *X*_*j*_ defined by kinesiology.

Finally, the potential of temporal relationship is calculated as follows:

(8) $$\begin{array}{c} \psi_{i}^{T}\left(X_{i}^{t},X_{i}^{t-1}\right)=p\left(X_{i}^{t}-X_{i}^{t-1};\mu_{i},\Sigma_{i}\right), \end{array}$$

where *μ*_*i*_ is the dynamics of *X*_*i*_ at the previous time step and Σ_*i*_ is a diagonal matrix with a diagonal element which is identical to |*μ*_*i*_|, similar to a Gaussian distribution with the time.

In this paper, the posterior distribution of model *X* conditioned on all input images up to the current joint *s* structure, the current time step *τ* and occlusion state variable ∧^1:*τ*^ are

(9) p(Xτ ∣ I1:τ;∧1:τ) =1Zexp{−∑i∈X1:τϕiC(I,Xi;λi)−∑ij∈EK1:τψijK(Xi,Xj)  −∑i∈ET1:τ,t∈1:τψiT(Xit,Xit−1)},

where *Z* is a normalization constant.

In other words, we put *ϕ*_*i*_, *ψ*_*ij*_^*K*^, *ψ*_*i*_^*T*^ into (4) and get body occluded joints positions:

(10) $$\begin{array}{c} {\hat{X}}^{t}=\underset{X^{t}}{\arg\;\max}\;p\left(X^{t}\;|\;I^{1:t};{\hat{\wedge }}^{t-1}\right), \end{array}$$

where *X* ^*t*^ is *X* joint location at *t* time.

The occluded relation among joints can be obtained by formula (2) as follows:

(11) $$\begin{array}{c} {\hat{\lambda }}_{i,j}^{t}=\underset{\wedge_{ij}\neq 0}{\arg\;\max\;}\phi_{i}\left(I^{t},{\hat{X}}_{i}^{t};\wedge_{ij}\right), \end{array}$$

where ${\hat{X}}_{i}^{t}$ is *X*_*i*_ position at *t* time.

Therefore, the occluded positions can be calculated by MRF at the entire time of motion.

## 3. Reconstruct the Missing Data of Point Trajectories

We use the SFM (structure from motion) model to reconstruct the missing trajectories of the occluded joints [27]. Consider a set of *P*_*n*_ point trajectories extracted from the *n* parts of human body that rigidly moves in *f* frames. By stacking each image trajectory in a single matrix *w*_*n*_ of 2*F* × *P*_*n*_, it is possible to express the global motion, which represents the complete trajectory. We define it as

(12) $$\begin{array}{c} w_{n}=M_{n}S_{n}=\left[\begin{array}{c} R_{1n}t_{1n}^{\prime }\\ \vdots \\ R_{Fn}t_{Fn}^{\prime } \end{array}\right]\left[\begin{array}{c} X_{1}\cdots X_{n}\\ Y_{1}\cdots Y_{n}\\ Z_{1}\cdots Z_{n}\\ 1\cdots 1 \end{array}\right], \end{array}$$

where *M*_*n*_ is the 2*F* × 4 human body motion matrix and *S*_*n*_ is the human body contour matrix in homogenous coordinates. Each frame-wise element *R*_*fn*_, for *f* = 1,…, *F*, is a 2 × 3 orthographic camera matrix that has to satisfy the metric constraints of the model (i.e., *R*_*fn*_ *R*_*fn*_^*T*^ = *I*_2  ×  2_). The 2-vector *t*_*Fn*_′ represents the 2D translation of the rigid object (in this paper, we consider the human body as a rigid object). We introduce the registered *w*_*n*_′ measurement matrix such that *w*_*n*_′ = *w*_*n*_ − *t*′1_*Pn*_^′*T*^, where 1_*Pn*_^′*T*^ is a vector of *P*_*n*_ ones and *t*′ = [*t*_1_^′*T*^,…, *t*_*F*_^′*T*^].

In the case of missing data due to occlusions, we define the binary mask matrix *G*_*n*_ of size 2*F* × *P*_*n*_ such that 1 represents a known entry and 0 denotes a missing one. In order to solve the components and thus the SFM problem, the equivalent optimization problem [28] can be defined as

(13) Minimize ||Gn⊗(wn−MnSn)||2,Subject  to RfnRfnT=I2  ×  2, f=1,…,F.

**Figure 1:**
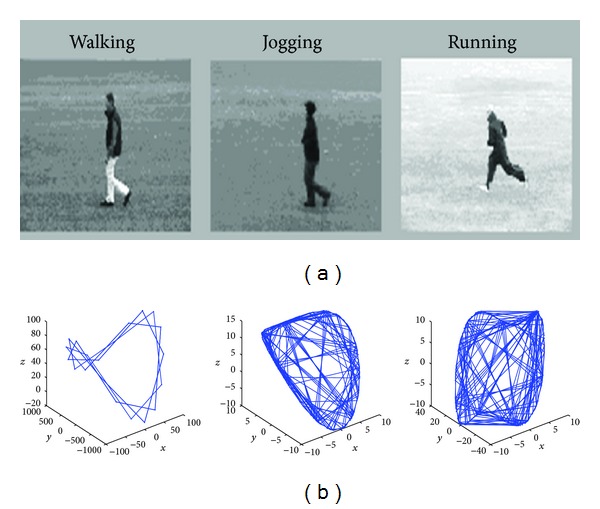
Examples of reconstructed 3-D trajectories. Hip rotations for walking, jogging, and running actions in the KTH dataset [30]. (a) It shows original images. (b) It shows reconstructing the missing data of point trajectories.

Therefore, we can get to make up for the missing point of the trajectory.  Figure 1 shows the reconstructed trajectory of the missing point.

## 4. Feature Representation

### 4.1. Motion Feature Extraction

The human action can be recognized in terms of hierarchical area model and relative velocity. In this paper, we use optical flow to detect the relative direction and magnitude of environmental motion observed in reference to an observer and also describe the movement of object from current image with respect to the last image. The optical flow [22, 29] equation can be assumed to hold for all pixels within a window centered at *p*, the local image flow (velocity) vector (*V*_*x*_, *V*_*y*_) must be satisfied, and we define some equations as follows.

(14) $$\begin{array}{c} I_{x}\left(s_{1}\right)V_{x}+I_{y}\left(s_{1}\right)V_{y}=-I_{t}\left(s_{1}\right),\\ I_{x}\left(s_{2}\right)V_{x}+I_{y}\left(s_{2}\right)V_{y}=-I_{t}\left(s_{2}\right),\\ \vdots \\ I_{x}\left(s_{d}\right)V_{x}+I_{y}\left(s_{d}\right)V_{y}=-I_{t}\left(s_{d}\right), \end{array}$$

where *s*_1_, *s*_2_,…, *s*_*d*_ are the pixels inside the window and *I*_*x*_(*s*_*i*_), *I*_*y*_(*s*_*i*_), and *I*_*t*_(*s*_*i*_) are the partial derivatives of the image *I* with respect to position *x*, *y* and time *t*, evaluated at the point *S*_*i*_ and the current time. These equations can be written in matrix form *Av* = *b*, where

(15) $$\begin{array}{c} A=\left[\begin{array}{c} I_{x}\left(s_{1}\right)I_{y}\left(s_{1}\right)\\ I_{x}\left(s_{2}\right)I_{y}\left(s_{2}\right)\\ \vdots \\ I_{x}\left(s_{n}\right)I_{y}\left(s_{n}\right) \end{array}\right],\;\;v=\left[\begin{array}{c} V_{x}\\ V_{y} \end{array}\right],\\ b=\left[\begin{array}{c} -I_{t}\left(s_{1}\right)\\ -I_{t}\left(s_{2}\right)\\ \vdots \\ -I_{t}\left(s_{d}\right) \end{array}\right]. \end{array}$$

The optical flow vector field *V*_*x*_ is further half-wave rectified into four nonnegative channels:

*F*_*x*_^+^, *F*_*x*_^−^, *F*_*y*_^+^, *F*_*y*_^−^, so that

(16) $$\begin{array}{c} F_{x}=F_{x}^{+}-F_{x}^{-},\;\;F_{y}=F_{y}^{+}-F_{y}^{-}. \end{array}$$

The motion descriptors of two different frames are compared using a version of the normalized correlation.

**Figure 2:**
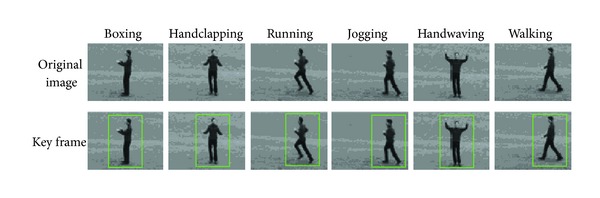
Results of key-frame extracted method.

Suppose the four channels for frame *i* of sequence *A* are *a*_1_, *a*_2_, *a*_3_, *a*_4_ and the four channels for frame *j* of sequence *B* are *b*_1_, *b*_2_, *b*_3_, *b*_4_; then the similarity between frame *A*_*i*_ and *B*_*i*_ is

(17) $$\begin{array}{c} S\left(A_{i},B_{i}\right)=\underset{t\in T\;}{\sum }\overset{4}{\underset{d=1\;}{\sum }}\underset{x,y\in I}{\sum }a_{c}^{i+t}\left(x,y\right)b_{b}^{j+t}\left(x,y\right), \end{array}$$

where *T* and *I* are the temporal and spatial extent of the motion descriptors. In this paper, we choose *T* = 20. Therefore, we can obtain the key frames by the frames cluster.  Figure 2 depicts results of key-frame extracted by the optical flow.

Then, we extract the feature from the key frame. We assume that the overall key frame number is *i*, the lengths of silhouette in horizontal and vertical are *h*(*i*) and *v*(*i*), respectively, and their ratio is *L*, so we define it as

(18) $$\begin{array}{c} L=\frac{v\left(i\right)}{h\left(i\right)},\\ v\left(i\right)=m_{\theta +(\pi /2)}\left(0,1\right)\Delta_{\theta +(\pi /2)}. \end{array}$$

We assume that this area has a maximum value in the *θ* direction.

**Figure 3:**
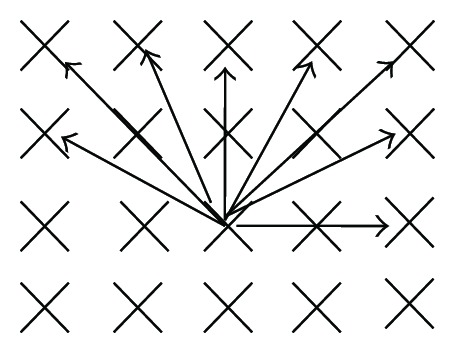
Eight searching directions in image.

The length of the projection area in the *θ* + (*π*/2) direction is defined as the area width:

(19) $$\begin{array}{c} h\left(i\right)=\frac{\max_{\theta }\left\{m_{\theta }\left(0,1\right)\Delta_{\theta }\right\}}{\min_{\theta }\left\{m_{\theta }\left(0,1\right)\Delta_{\theta }\right\}}, \end{array}$$

where Δ is the distance of pixels [31], *B* is the binary image of the human body area, *m*_*θ*_(0,1) is the region *B* in the perpendicular direction to the parallel lines intersecting the number of lines in group, and Δ_*θ*_ is the distance between the scan lines. In this paper, we use the eight search directions as shown in Figure 3. Therefore, distances among search lines, respectively, are

(20) Δ0=Δπ/2=Δ,Δπ/4=Δ3π/4=Δ2Δarctg(1/2)=Δarctg2=Δ(π/2)+arctg(1/2)=Δ(π/2)+arctg2=Δ5.

**Figure 4:**
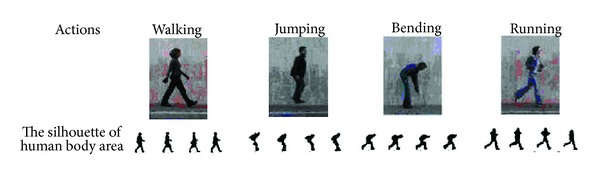
The silhouette of human body area.

In order to implement the silhouette of human body area, a series of steps must be followed:

1. using Gaussian filter to eliminate any noise in video sequence;
2. using filter to find the edge strength, which estimates the gradient in the *x*-direction and the other estimating the gradient in the *y*-direction;
3. the direction of the edge is computed using the gradient in the *x* and *y* directions;
4. after the edge directions are known nonmaximum suppression now has to be applied. Nonmaximum suppression is used to trace along the edge in the edge direction and suppress any pixel value (sets it equal to 0) that is not considered to be an edge. This will give a thin line in the output;
5. threshold *T*1 is applied to the frames, and an edge has an average strength equal to *T*1. Any pixel in the frame that has a value greater than *T*1 is presumed to be an edge and is marked as such immediately and then any pixels that are connected to this edge pixel and that have a value.  Figure 4 shows the silhouette of human body area.

Therefore, we can obtain the body contours by this step and use the optical flow descriptor and *L* descriptor (the ratio of the width and height) to represent the video frames. To construct the codebook, we randomly select a subset from all the frames and compute the affinity matrix on this subset of frames, where each entry in the affinity matrix is the similarity between frame *i* and frame *j* calculated using the normalized correlation described above. Then, we run *k*-medoid clustering on this affinity matrix to obtain *V* clusters. Code-words are then defined as the centers of the obtained clusters.

### 4.2. Construct the Codebook

In the end, each video sequence is converted to the “bag of words” representation by replacing each frame by its corresponding codeword and removing the temporal information.

We follow the instruction from statistical text document analysis, and each image is represented as a bag of code-words. Given a training set of images with annotation words, we use the following notation. Each image is a collection of *M* visual feature code-words, denoted as *v* = {*v*_1_ : *M*}, where *v*_*m*_ is a unit-basis vector of size *V*_*s*_ with exactly one nonzero entry representing the index of current visual feature in the visual feature dictionary of size *V*_*s*_. Similarly, for one image annotated with *N* words *W* = {*w*_1_ : *N*}, we denote each word *w*_*n*_ as a unit-basis vector of size *V*_*t*_ again with only one taking values 1 and 0 otherwise; here *V*_*t*_ is the word dictionary size. Therefore, a collection of *D* training image word pairs can be denoted as {*v*_1:*D*_, *W*_1:*D*_}.

## 5. Action Classification

In order to capture the correlation of topics, we model the hyperparameter of topic prior distribution as multivariate normal distribution instead of Dirichlet, similar to [29, 32] and the structure topics of dependencies by covariance matrix, and then we use the logistic normal function:

(21) $$\begin{array}{c} f\left(\theta_{i}e\right)=\left(\frac{\exp\theta_{i}}{\sum_{j=1}^{K}\exp\theta_{j}}\right) \end{array}$$

to project the multivariate normal to topic proportions for each image; here *K* is the topic number.

Let {*μ*, Σ} be a *K-*dimensional mean and variance matrix from normal distribution, and let topics *π*_1:*k*_ be *K* multi-nomials over fixed vocabulary with size *V*_*s*_ and let *β*_1:*K*_ be *K* multi-nomials over a fixed text word vocabulary with size *V*_*t*_. The CTM generates an image-word pair with *M* image code-words and *N* annotation words [29, 32] from the following generative process:

1. draw topic proportions *θ*{*μ*, Σ} ~ *N*(*μ*, Σ)
2. for each visual feature *v*_*m*_, *m* ∈ {1,…, *m*}
  - draw topic assignment *z*_*m*_ ~ Mult(*f*(*θ*))
  - draw visual feature *v*_*m*_ ~ Mult(*π*_*z*_*m*__)
3. for each textual word *w*_*n*_, *n* ∈ {1, ,…, *N*}
  - draw feature index *y*_*n*_ ~ Unif(1, ,…, *M*)
  - draw textual word *w*_*n*_ ~ Mult(*β*_*z*_*y*_*n*___).

**Figure 5:**
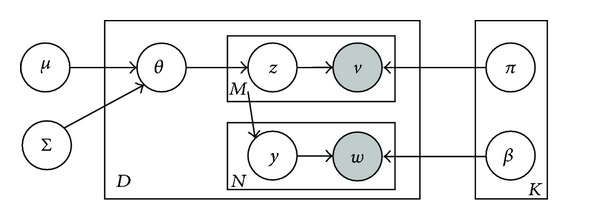
Graphical representation of the variational distribution for CTM.

Firstly, we generate *M* feature *v*_*m*_ from correlated topic proportions *θ*, conditional on the topic feature multinomial *π*, then for each of the *N* text words, one of the *M* features is selected and correspondingly assigned to a text word *w*_*n*_, conditional on the topic word multinomial *β*. This model is shown as a directed graphical model in Figure 5.

From the generative process of CTM, it could be learned that topic correlations are modeled and generated through the covariance matrix Σ of prior multivariate normal distribution. To learn the parameters of CTM that maximizes the likelihood of training data, we iteratively estimate the model parameters of latent variables. In this paper, the first step is to add and calculate a set of variational parameters to obtain the approximate lower-bound on likelihood of each sample. The latter step is to estimate the model parameters that maximize the log likelihood of the whole training samples. In the graphical representation of CTM in Figure 5, *θ* is conditional dependent on the parameter *π*, which leads to intractability for computing the log likelihood. The mean-field variational distribution is

(22) q(θ,z,y)=q(θ ∣ γ,v)∏m=1Mq(Zm ∣ ϕm)∏n=1Nq(yn ∣ λn),

where (*γ*, *v*) is variational mean and variance of normal distribution, *ϕ*_*m*_ is a variational multinomial over *K* topics, and *λ*_*n*_ is a variational multinomial over codewords.

Let Δ = {*μ*, Σ, *π*, *β*} denote model parameters; we bound the log-likelihood of an image-annotation pair (*v*, *w*) as follows:

(23) logp(v,w ∣ Δ) =log∫θp(v,w,θ,z,y ∣ Δ)q(θ,z,y)q(θ,z,y)dx dy ≥Eq[logp(v,w,θ,z,y ∣ Δ)]  −Eq[logq(v,w,θ,z,y ∣ Δ)]=ι(γ,v,ϕ,λ;Δ),

where *E*_*q*_ is the expectation according to the variational distribution. Taking the log likelihood function *ι*(*γ*, *v*, *ϕ*, *λ*; Δ) as object function, we fit these parameters with coordinate ascent to maximize the object function:

(24) ι=Eq[logp(θ ∣ μ,Σ)]+∑mEq[logp(zm ∣ θ)] +Eq[logp(vm ∣ zm,π)]+∑mEq[logp(ym ∣ M)] +Eq[logp(w ∣ ym,zm,β)]−Eq[logq(θ ∣ γ,v)] −∑mEq[logq(zm ∣ θ)]−∑nEq[logq(ym ∣ λ)],

(25) ∂ι(γ)∂γ=−∑−1(γ−μ)+∑m=1Mϕm,1:K −(Mς)exp(γ+ν22),

(26) $$\begin{array}{c} \frac{\partial \iota \left(\nu_{i}\right)}{\partial \nu_{i}^{2}}=-\frac{\sum_{ii}^{-1}\;}{2}-\left(\frac{M}{2\varsigma }\right)\exp\left(\gamma +\frac{\nu_{i}^{2}}{2}\right)+\frac{1}{\left(2\nu_{i}^{2}\right)}. \end{array}$$

Then, we update variational multinomial *ϕ*. The terms in *ι* with respect to *ϕ*_*m*_ are

(27) $$\begin{array}{c} \iota \left(\phi_{m}\right)=\overset{K}{\underset{i=1}{\sum }}\phi_{mi}\left(\gamma_{i}+\log\pi_{i,v_{m}}-\log\phi_{mi}+\overset{N}{\underset{n=1}{\sum }}\lambda_{nm}\log\beta_{i,w_{n}}\right), \end{array}$$

(28) $$\begin{array}{c} \frac{\partial \iota \left(\phi_{mi}\right)}{\partial \phi_{mi}}=\gamma_{i}+\log\pi_{i,v_{m}}+\overset{N}{\underset{n=1}{\sum }}\lambda_{nm}\beta_{i,w_{n}}-\log\phi_{mi}+C, \end{array}$$

where ∑_*i*=1_^*K*^ *ϕ*_*mi*_ = 1 is the multinomial constrain. *C* is the constant.

The full variational inference procedure repeats the updates of (24), (25) until (23); the object function converges.

Then, we will obtain the parameter estimation. Given a collection of human action image data with annotation words {*v*_*d*_,*w*_*d*_}_*d*=1_^*D*^, we find the maximum likelihood estimation for parameter {*μ*, Σ, *π*, *β*}.

We define Δ = {*μ*, Σ, *π*, *β*}; the overall log likelihood of collection is bounded by

(29) $$\begin{array}{c} \iota =\overset{D}{\underset{d=1}{\sum }}\log p\left(v_{d}w_{d}\;|\;\Delta \right)\geq \overset{D}{\underset{d=1}{\sum }}\iota_{d}\left(\gamma_{d},v_{d},\phi_{d},\lambda_{d};\Delta \right). \end{array}$$

We maximize the lower boundary of *ι*, by plugging (23) into (17), and then update model parameters by setting derivation equal zero with respect to each model parameter.

The terms containing *π*_*ij*_ are

(30) $$\begin{array}{c} \iota \left(\pi_{ij}\right)=\overset{D}{\underset{d=1\;}{\sum }}\overset{M_{d}}{\underset{m=1\;}{\sum }}\overset{K}{\underset{i=1\;}{\sum }}\overset{V_{s}}{\underset{j=1}{\sum }}\phi_{dmi}\log\pi_{i,v_{m}}-C\left(\overset{V_{s}}{\underset{j=1}{\sum }}\pi_{ij}-1\right). \end{array}$$

Setting ∂*ι*(*π*_*ij*_)/∂*π*_*ij*_ = 0 leads to

(31) $$\begin{array}{c} \pi_{ij}^{\prime }\propto \overset{D}{\underset{d=1\;}{\sum }}\overset{M_{d}}{\underset{m=1}{\sum }}\phi_{dmi}v_{dm}^{j}. \end{array}$$

The terms containing *β*_1:*K*_ are

(32) ι(βi,j)=∑d=1 D∑n=1 Nd∑i=1 K∑j=1 Vt∑m=1Mdϕdmiλnmlogβi,wn −C(∑j=1Vtβij−1).

Setting ∂*ι*(*β*_*ij*_)/∂*β*_*ij*_ = 0 leads to

(33) $$\begin{array}{c} \beta_{ij}^{\prime }\propto \overset{D}{\underset{d=1\;}{\sum }}\overset{N_{d}}{\underset{n=1}{\sum }}w_{dn}^{j}\overset{M_{d}}{\underset{m=1}{\sum }}\phi_{dmi}\lambda_{dnm}. \end{array}$$

The terms containing *μ* are

(34) $$\begin{array}{c} \iota \left(\mu \right)=-\left(\frac{1}{2}\right)\overset{D}{\underset{d=1}{\sum }}{\left(\gamma_{d}-\mu \right)}^{T}\overset{-1}{\underset{\;}{\sum }}\left(\gamma_{d}-\mu \right). \end{array}$$

Setting ∂*ι*(*μ*)/∂*μ* = 0 leads to

(35) $$\begin{array}{c} \mu^{\prime }=\left(\frac{1}{D}\right)\overset{D}{\underset{d=1}{\sum }}\gamma_{d}. \end{array}$$

The terms containing Σ are

(36) ι(Σ)=∑d=1D{(12)log|σ−1|−(12)Tr[diag(vd2)Σ−1] −(12)(γd−μ)TΣ−1(γd−μ)}.

Setting ∂*ι*(Σ)/∂Σ = 0 leads to

(37) $$\begin{array}{c} \Sigma^{\prime }=\left(\frac{1}{D}\right)\overset{D}{\underset{d=1}{\sum }}\left(I\times v_{d}^{2}+\left(\gamma_{d}-\mu^{\prime }\right){\left(\gamma_{d}-\mu^{\prime }\right)}^{T}\right). \end{array}$$

If we obtain variational parameters {*γ*_*d*_,*v*_*d*_,*ϕ*_*d*_,*λ*_*d*_,*ς*_*d*_}_*d*=1_^*D*^ for all the training samples, we can update the model parameters {*μ*, Σ, *π*, *β*} by plugging them into (30), (34), and (36) until the overall likelihood in (28) converges. Therefore, We approximate the conditional distribution of code-words as follows:

(38) $$\begin{array}{c} p\left(w\;|\;v\right)=\overset{M}{\underset{m=1}{\sum }}\underset{z_{m}}{\sum }q\left(z_{m}\;|\;\phi_{m}\right)p\left(w\;|\;z_{m},\beta \right). \end{array}$$

The conditional probability *p*(*w* | *v*) can be treated as the predicted confidence score of each annotation word in word vocabulary, given the whole code-words of the unknown human behavior.

## 6. Experimental Result

### 6.1. Datasets

We test our algorithm on three datasets: the Weizmann human motion dataset [33], the KTH human action dataset [30], and the UIUC action dataset [34]. All the experiments are conducted on a Pentium 4 machine with 2 GB of RAM, using the implementation on MATLAB. The dataset and the related experimental results are presented in the following sections.

The KTH dataset is provided by Schuldt which contains 2391 video sequences with 25 actors showing six actions. Each action is performed in 4 different scenarios.

The WEIZMANN dataset is provided by Blank which contains 93 video sequences showing nine different people, each performing ten actions, such as run, walk, skip, jump-jack, jump-forward-on-two-legs, jump-in-place-on-two-legs, gallop-sideways, wave-two-hands, wave-one-hand, and bend.

The UIUC action dataset is created by the University of Illinois at Urbana-Champaign (UIUC) in 2008 for human activity recognition. The activities are walking, running, jumping, waving, jumping jacks, clapping, jumping from sit up, raising one hand, stretching out, turning, sitting to standing, crawling, pushing up, and standing to sitting.

For every dataset, 12 video sequences are taken by four subjects (out of the five) used for training and the remaining three videos for testing. The experiments are repeated five times. The performance of different methods is shown using the average recognition rate. In order to evaluate the performance of action recognition, we report the overall accuracy on three datasets.

### 6.2. Comparison

**Figure 6:**
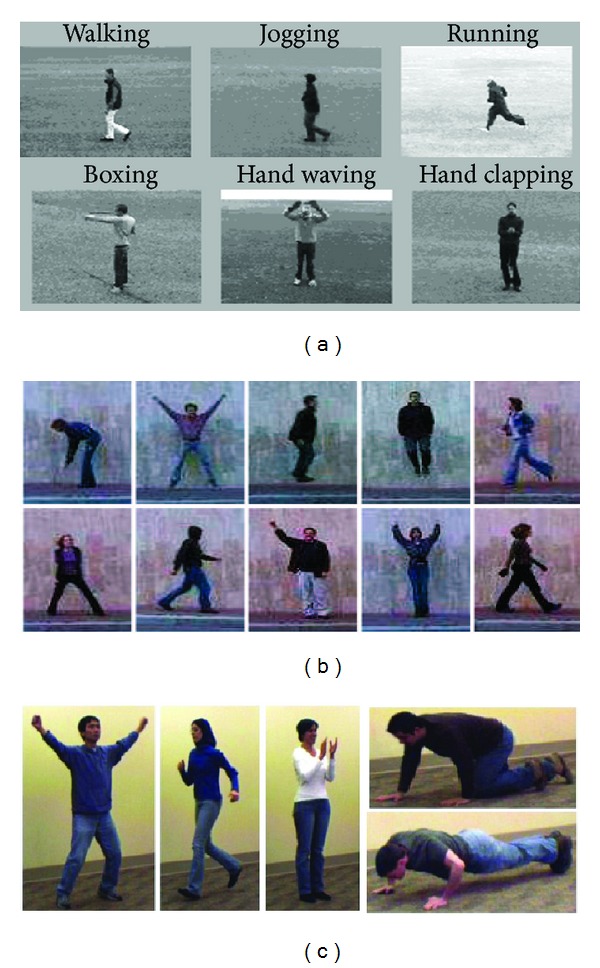
Sample frames from our datasets. The action labels in each dataset are as follows: (a) KTH dataset: walking (a1), jogging (a2), running (a3), boxing (a4), handclapping (a5); (b) Weizmann dataset: running, walking, jumping-jack, waving-two-hands, waving-one-hand, bending; (c) UIUC action dataset: raising two hands (a1), running (a2), clapping (a3), stretching out (a4), and pushing up (a5).

**Figure 7:**
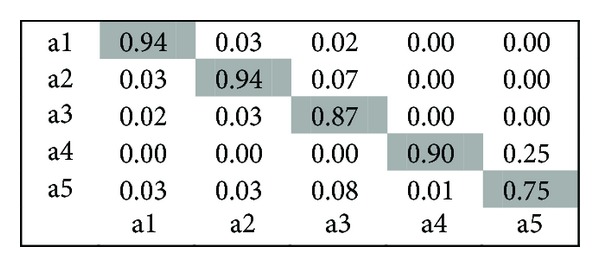
Confusion matrix for KTH dataset.

*KTH Dataset.* It contains six types of human actions (walking, jogging, running, boxing, hand waving, and hand clapping) performed several times by 25 subjects in four different scenarios: outdoors, outdoors with scale variation, outdoors with different clothes, and indoors. Representative frames of this dataset are shown in Figure 6(a). After process of restoring missing coordinate position, we use the proposed method, and the classification results of KTH dataset obtained by this approach are shown in Figure 7 and indicate quite a small number of videos are misclassified, particularly, the actions “running” and “handclapping” which more tend to be confused.

**Figure 8:**
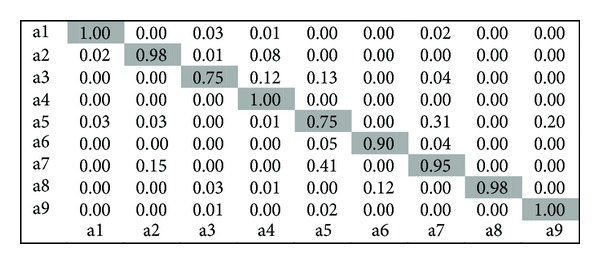
Confusion matrix for Weizmann dataset.

*The Weizmann Dataset.* The Weizmann human action dataset contains 83 video sequences showing nine different people and all performing nine different actions: bending (a1), jumping jack (a2), jumping forward on two legs (a3), jumping in place on two legs (a4), running (a5), galloping sideways (a6), walking (a7), waving one hand (a8), and waving two hands (a9). The figures were tracked and stabilized by using the background subtraction masks that come with this dataset. Some sample frames are shown in Figure 6(b). The classified results achieved by this approach are shown in Figure 8. 

**Figure 9:**
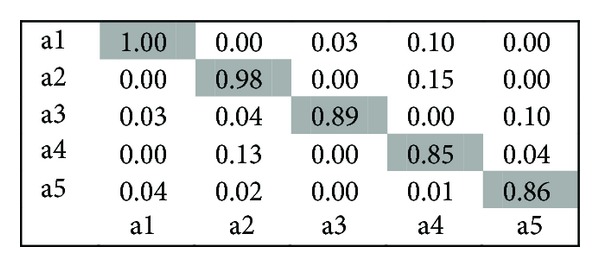
Confusion matrix for UIUC dataset.

*The UIUC Action Dataset.* This dataset consists of 532 high resolution sequences of 14 activities performed by eight actors. The activities are walking, running, jumping, waving, jumping jacks, clapping, jumping from sit-up, raising one hand, stretching out, turning, sitting to standing, crawling, pushing up, and standing to sitting. Some sample frames are shown in Figure 6(c). The classified results achieved by this approach are shown in Figure 9.

In this paper, we identify jogging, running, walking, and boxing and compare the proposed method with the four state-of-the-art methods in the literature: Zhang and Gong [35], Gong et al. [36], and Chang et al. [37] in three datasets. As shown in Tables 1, 2, and 3, the existing methods, the low recognition accuracy because these action are not only occlusion situation are complex, but also the legs have complex beat, motion and other group actions. The proposed method can overcome these problems, and the recognition accuracy and average accuracy are higher than the comparative method.

The experimental results show that the approach proposed in the paper can get satisfactory results and significantly performs better comparing the average accuracy with that in [35–37] because of a practical method adopted in the paper.

## 7. Conclusions and Future Work

In this paper, we proposed an adaptive occlusion action recognition method for human body movement. Our method successfully recognizes without assuming a known and fixed depth order. We have presented the MRF model and SFM model, which estimates the adaptive occlusion state and recovers the important missing parts of objects in a video clip. This paper presents a new method of human self-occlusion behavior recognition, which is based on optical flow and correlated topic model (CTM). Then, we have employed the optical flow motion feature to extract the key frame and calculated the ratio of the width and height from human silhouette. Finally, we use the topic model of correlated topic model (CTM) to classify and recognize action. Experiments were performed on the KTH, Weizmann, and UIUC action datasets to test and evaluate the proposed method. The compared experiment results showed that the proposed method was more effective than compared methods and better than other approaches [35–37].

Future work will deal with adding complex event detection to the proposed system, involving more complex problems such as dealing with more variable motion, interperson occlusions, and possible appearance similarity of different people and increasing the database size.

## Figures and Tables

**Table 1:** Compared with other approaches on KTH dataset.

Method	Average recognition rate (%)
The proposed method	90.60
Zhang and Gong [35]	83.40
Gong et al. [36]	89.20
Chang et al. [37]	88.03

**Table 2:** Compared with other approaches on Weizmann dataset.

Method	Average recognition rate (%)
The proposed method	89.20
Zhang and Gong [35]	85.30
Gong et al. [36]	86.30
Chang et al. [37]	82.10

**Table 3:** Compared with other approaches on UIUC dataset.

Method	Average recognition rate (%)
The proposed method	93.30
Zhang and Gong [35]	89.70
Gong et al. [36]	92.50
Chang et al. [37]	91.20
